# Supplementary material for: Selective excitation of vibrations in a single molecule
Source: Nat Commun. 2024 Aug 14;15:6983. doi: 10.1038/s41467-024-51419-1 (PMC11324655; doi:10.1038/s41467-024-51419-1)
Supplement: Supplementary file 1 — Supplementary Information [file 41467_2024_51419_MOESM1_ESM.pdf]

Supplementary Information for

Selective Excitation of Vibrations in a Single Molecule

Yang Luo[1,*], Shaoxiang Sheng[1], Michele Pisarra[2,3], Alberto Martin-Jimenez[1,4], Fernando Martin[4,5]*, Klaus Kern[1,6], Manish Garg[1,*]

[1] Max Planck Institute for Solid State Research, Heisenbergstr. 1, 70569 Stuttgart, Germany
[2] Dipartimento di Fisica, Università della Calabria, Via P. Bucci, cubo 30C, 87036, Rende (CS), Italy
[3] INFN-LNF, Gruppo Collegato di Cosenza, Via P. Bucci, cubo 31C, 87036, Rende (CS), Italy
[4] Instituto Madrileño de Estudios Avanzados en Nanociencia (IMDEA Nano), Faraday 9, Cantoblanco, 28049 Madrid, Spain
[5] Departamento de Química, Módulo 13, Universidad Autónoma de Madrid, 28049 Madrid, Spain
[6] Institut de Physique, Ecole Polytechnique Fédérale de Lausanne, 1015 Lausanne, Switzerland

*Authors to whom correspondence should be addressed.

y.luo@fkf.mpg.de, fernando.martin@imdea.org and mgarg@fkf.mpg.de

Contents

Section I. Fluorescence and Raman scattering processes in a molecule 2
Section II. Density functional theory calculations 4
Section III. Optical setup for the single-molecule spectroscopy 7
Section IV. Comparison between the experimental and the calculated Raman spectra 8
Section IV. UV-Vis absorption spectrum of H_2Pc molecules 10
References 11

Section I. Fluorescence and Raman scattering processes in a molecule

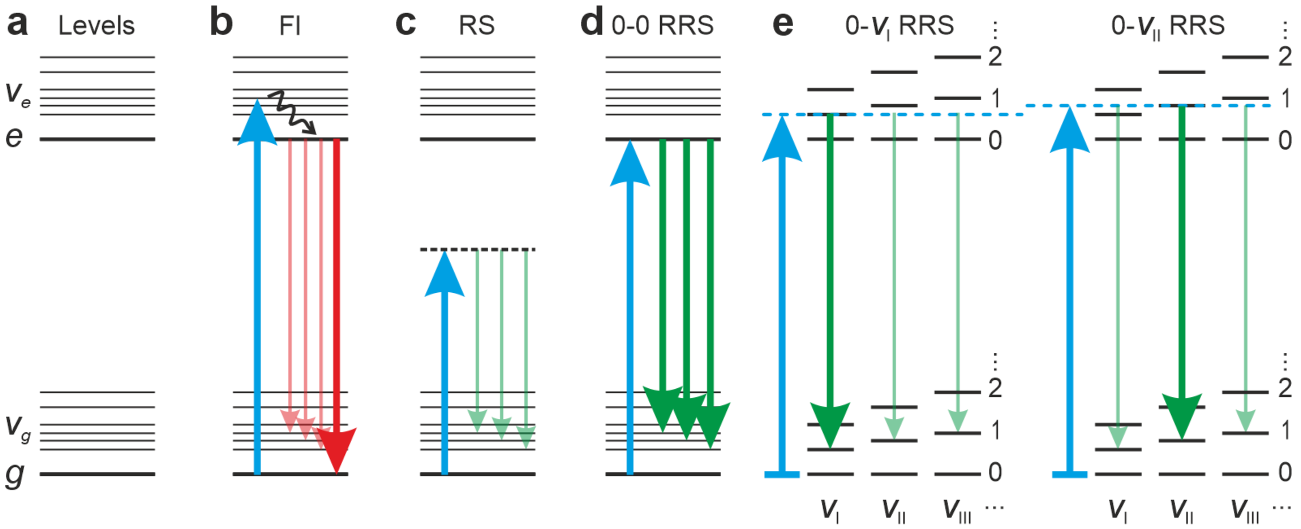

Supplementary Fig. 1: Schematic illustration of various processes ensuing photoexcitation in a molecule. **a,** The energy alignment of various vibrational modes associated with the ground (g) and excited (e) electronic states. **b, c, d,** Depiction of fluorescence (Fl), Raman scattering (RS), and resonance Raman scattering (RRS) processes in the molecule, respectively. The blue upward arrows indicate the tunable laser exciation, the black wave arrow indicated the vibrational relaxation, The red downward arrows indicate the fluorescence processes, and the blue downward arrows indicate the Raman transitions. The relative intensities of different transitions are symbolized by the thickness of the downward arrows. **e,** Schematic description of vibrationally resolved resonance Raman scattering processes involving, the ν_{I} (left panel) and ν_{II} (right panel) vibrational modes of the ground electronic state, g. Numerals, 0, 1, 2… represent the quantum numbers of the various vibrational modes (e.g. ν_{I} or ν_{II}) in the two electronic states. The molecule is initially in the vibrational ground state before the laser excitation.

Many processes ensue after the photoexcitation of a molecule. We start by considering the interaction of the light with the quantized electronic and vibrational levels of a single molecule, which is simplified as the energy levels as shown in Supplementary Fig. 1a. The electronic ground and the excited states are marked by g and e, respectively. The vibrational levels in the two electronic states are denoted by ν_g and ν_e. Here, three light interaction processes are considered: fluorescence (Fl), Raman scattering (RS), and the resonance Raman scattering (RRS).

In the fluorescence (Fl) process as depicted in Supplementary Fig. 1b, an incident photon excites the system to one of the $|\nu_e, e\rangle$ levels. The system can redistribute the excess vibrational energy among its various modes by internal conversion or by exchanging energy with its environment to reach the bottom of the excited electronic state, i.e., $|0, e\rangle$. This state can then generate the fluorescence emission by the radiative transition to the ground electronic state. Several emission pathways are possible as indicated by red downward arrows. If

the geometry molecule is similar in the ground and excited electronic states, the most intense transition (thick arrow) happens between the $|0, e\rangle$ and $|0, g\rangle$ states.

In the Raman Scattering (RS) process, the incident light is inelastically scattered by interacting with a vibrational mode of the molecule. The process can be sketched with the incident photon absorbed to a virtual state and then re-emitted with a lower energy, as shown in Supplementary Fig. 1c. For simplicity we consider only Stokes processes, which are relevant for the current experiment. Multiple Raman scatterings are possible and the energy loss of the scattered light depends on the energy of the excited vibrational modes. The Raman scattering probability is usually very low, as symbolized by the thin downward green arrows in Supplementary Fig. 1c.

The Raman scattering cross-section can increase dramatically if the incident light is resonant with an electronic transition in the molecule. This phenomenon is depicted in Supplementary Fig. 1d, where the incident light is resonant with the electronic transition between the $|0, g\rangle$ and $|0, e\rangle$ states. This electronic transition is also called the 0-0 transition, because it involves the vibrational ground states of the g and e electronic states. In this case, all the Raman modes coupled to the excited state are enhanced, as symbolized by the thick downward green arrows.

The energy of the incident light can be tuned to be resonant with the $|0, g\rangle$ and $|v_{\mathrm{I}}, e\rangle$ states, as shown in the left panel of Supplementary Fig. 1e. While all the Raman scattering processes from $|v_{\mathrm{I}}, e\rangle$ towards $|v_{\mathrm{I}}, g\rangle$, $|v_{\mathrm{II}}, g\rangle$, $|v_{\mathrm{III}}, g\rangle$,... are possible (thin downward green arrows), the one towards the $|v_{\mathrm{I}}, g\rangle$ state will be dramatically enhanced (thick downward green arrow) because of the dominance of the Frank-Condon factor $\langle v_{\mathrm{I}}, e|v_{\mathrm{I}}, g\rangle$, provided that the geometry of the molecule in the electronic e and g states is not very different. A similar scenario involving the $|v_{\mathrm{II}}\rangle$ vibrational mode is depicted in the right panel of Supplementary Fig. 1e, when the energy of the incident light is tuned to be resonant with the $|0, g\rangle$ and $|v_{\mathrm{II}}, e\rangle$ states. Due to the narrow Raman linewidths in the single-molecule spectrum, distinguishing the resonance Raman transitions from different vibronic transitions is possible, as sketched in Supplementary Fig. 1e. Hence, by modulating the photon energy of the incident light, it is possible to selectively populate a specific vibrational mode in the ground state. In the vibrationally resolved resonant Raman scattering processes, different laser excitation photon energies will result into different enhancement factors of the Raman scattering rates. In all the cases, the most prominent Raman scattering occurs between identical vibrational modes in the two electronic states (thick green arrows in Supplementary Fig. 1e), i.e. when their energy difference will be almost identical to that of the fluorescence transition (thick red arrow in Supplementary Fig. 1b).

Section II. Density functional theory calculations

Atomistic calculations have been performed with the Gaussian 16 program package[1]. We carried out unrestricted all electron calculations using the B3LYP functional[2] and the 631G(d,p) basis function. The resonance Raman spectroscopy (RRS) calculations have been carried out taking into account the excited state involved in the transition as implemented in the Gaussian 16 package[3,4]. We have conducted the simulations for both the H_2Pc and the HP_C^- molecules, and the results for the HP_C^- molecule are described here in detail.

We optimized the geometry of the molecule in the ground state, obtaining also the vibrational spectrum. The Raman intensities of the ground electronic state are also obtained during this step. The ground electronic state of the HP_C^- molecule is characterized by a flat geometry, as shown in the left panel of Supplementary Fig. 2. The vertical electronic excitation spectrum and transition dipole moments at the ground state geometry were calculated within a TD-DFT approach obtaining up to the 20th transition (see Supplementary Table 1). This procedure allowed us to identify the electronic transitions with nonzero oscillator strength. The S1 and S2 excited states correspond to the experimentally electronic states named Q_x and Q_y[5,6]. In our case, the relevant excited state is the singlet state S1 (the third overall excited state, highlighted in red in Supplementary Table 1), which contributes to the fluorescence peaks in Fig. 1b of the main text. A geometry optimization was then performed for the molecules in the excited electronic S1, using the ground state geometry as the starting point. We first found a transition state, with one imaginary frequency in the vibrational spectrum, characterized by a flat arrangement of the atoms (S1-I in Supplementary Fig. 2). A further optimization of the geometry, with adding little displacements to the S1-I configuration, leads to the equilibrium configuration in the excited state (S1-F in Supplementary Fig. 2), which is characterized by a bent geometry.

Supplementary Table 1: HP_C^- vertical transitions at the ground electronic state geometry.

Ex. State	Ex. State #	Spin mult.	Energy (eV)	Energy (nm)	Oscillator Strength
T1	1	3	1.2364	1002.8	0
T2	2	3	1.2926	959.22	0
**S1**	**3**	**1**	**2.1365**	**580.3**	**0.3634**
S2	4	1	2.156	575.07	0.3677
T3	5	3	2.414	513.61	0
T4	6	3	2.4279	510.67	0
T5	7	3	2.6629	465.6	0
T6	8	3	2.7088	457.7	0
S3	9	1	2.83	438.11	0
S4	10	1	2.8359	437.2	0.0001
T7	11	3	2.8755	431.17	0
T8	12	3	2.8902	428.98	0
T9	13	3	2.9519	420.02	0
T10	14	3	3.003	412.87	0
T11	15	3	3.0066	412.37	0
T12	16	3	3.1949	388.06	0
T13	17	3	3.2201	385.03	0

S5	18	1	3.2202	385.02	0.1869
T14	19	3	3.245	382.07	0
S6	20	1	3.2965	376.1	0.1772

In the resonance Raman scattering calculation we took into consideration both the S1-F and the S1-I geometries as intermediate states. In the cases of the S1-F configuration, the overlap integral between the vibrational ground states gave very small values, due to the large differences in the geometries of the ground and the excited state. For this reason, we continued our analysis using the S1-I configurations, neglecting the first (imaginary) vibrational mode. The RRS calculation has been carried out within a time-independent approach, as implemented in the Gaussian software, using various incident light frequencies. Since the S1->S0 transitions are dipole-allowed, the calculation included the Frank-Condon terms only, while the second order Herzberg-Teller terms of the dipole matrix element expansion were not included. We assumed a 40 cm^{-1} broadening for the incident light and a 10 cm^{-1} broadening for the scattered Raman signal in the calculations.

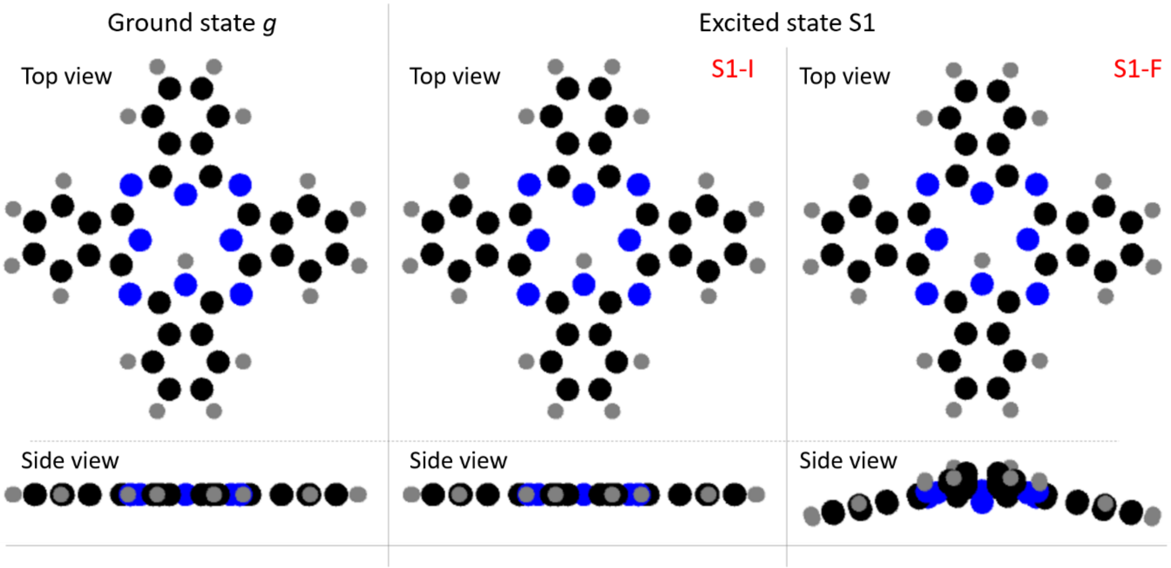

Supplementary Fig. 2: Geometry optimization of the HP_C^- molecule in the ground state (*g*, left panel) and the S1 excited electronic state (central and right panels). The S1-I geometry corresponds to the atomic configuration of the transition state, with one imaginary frequency; the S1-F geometry shows the equilibrium configuration in the excited state. The C, N, and H atoms are rendered as black, Blue and Gray disks, respectively.

Supplementary Fig. 3 shows the calculated resonance Raman scattering spectra for different photon energies of the incident light. To ease comparison with Fig. 2a of the main text, the spectra are plotted with their x-axis being the relative energy (in meV) with respect to the fluorescence emission peak in the range from −25 to 25 meV. Several peaks of different intensities can be clearly identified in the spectra. An apparent gradual shift of the narrow emission peaks is observed upon increasing the photon excitation energy of the laser, which is

consistent with the experimental observations. The intensity of the Raman peaks is greatly enhanced when their transition energy approaches the energy of the 0-0 transition, whose spectral position is annotated by the vertical dashed red line in the plot.

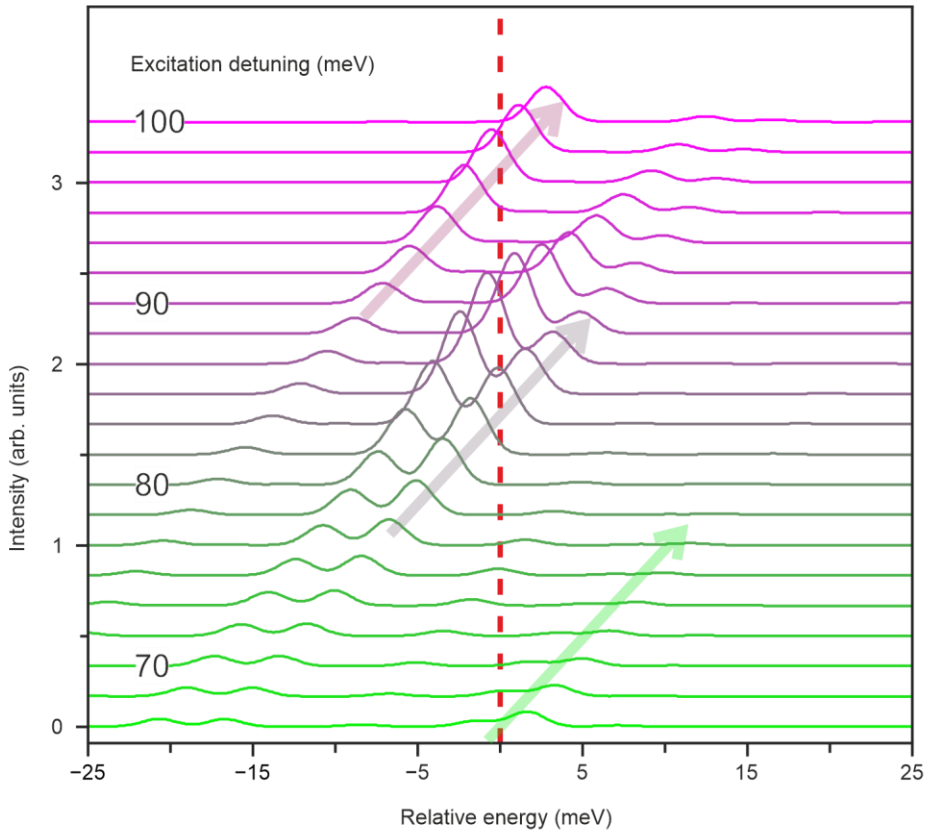

Supplementary Fig. 3: Calculated resonance Raman spectra for different photon energies of the incident light. The energy detuning between the incident photons and the calculated optical gap is annotated on the left side of the spectra. The resonance Raman spectra are plotted with their x-axis being the relative energy (in meV) with respect to the 0-0 transition (fluorescence peak) at 616.05 nm (red dashed line). The spectra are displaced vertically for clarity.

Section III. Optical setup for the single-molecule spectroscopy

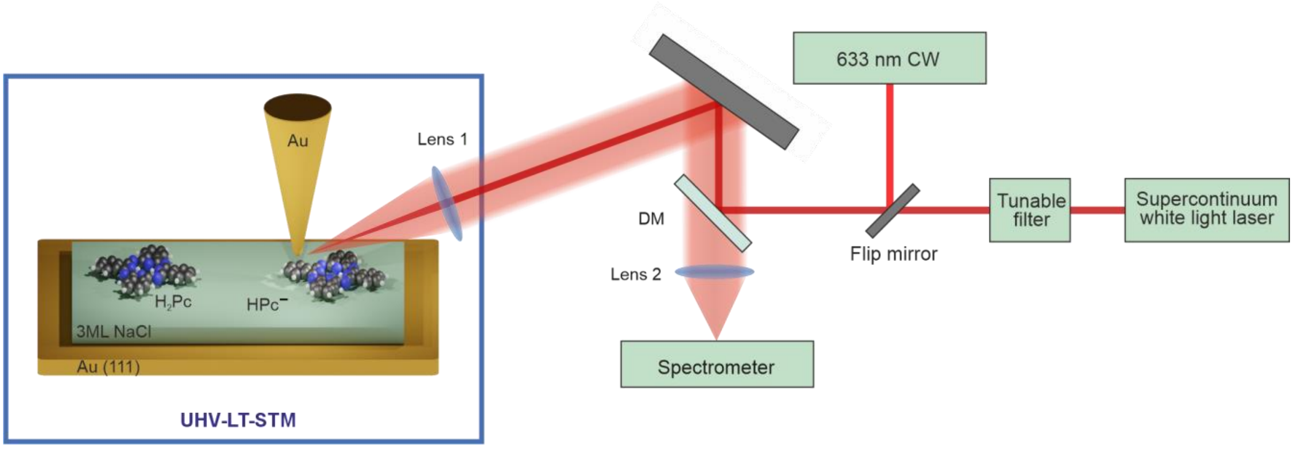

Supplementary Fig. 4: Optical setup for the single-molecule spectroscopy. Both the CW laser centered at 633 nm and the ps-laser with tunable excitation wavelength can be used as the excitation laser beam. A flip mirror is used to switch between the excitation sources of the CW laser and the ps-laser. DM: dichroic mirror.

Section IV. Comparison between the experimental and the calculated Raman spectra

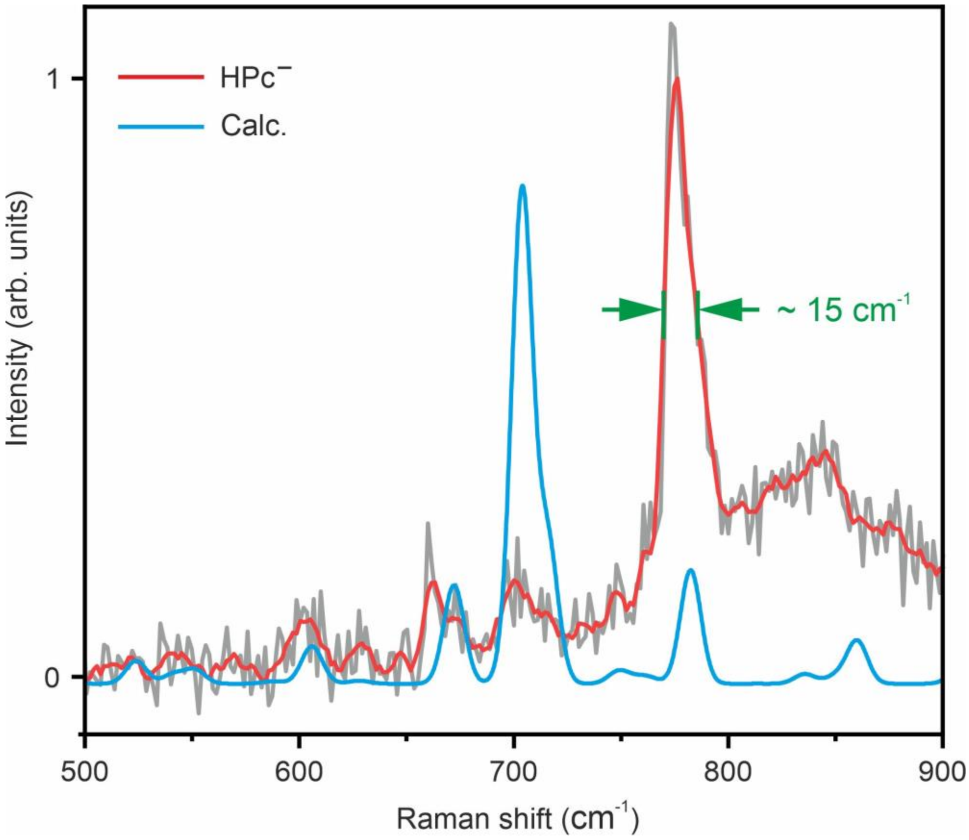

Supplementary Fig. 5: Comparison between the experimental TEPL spectrum from HPc^{-} molecule (red curve) and the calculated Raman spectrum for the ground electronic state (blue curve). The TEPL spectrum is adapted from Fig. 1 in the main-text, which was recorded with CW laser excitation (λ ~ 633 nm and laser power of 0.2 mW) at $V = -1$ V, $I = 4$ pA, and $t = 10$ s. The most prominent vibrational peak comes from the resonance Raman scattering, while the other vibrational modes are still visible in the tip-enhanced Raman spectroscopy. The spectral resolution of TEPL spectra measured with CW laser excitation is estimated to be ~ 15 cm^{-1} (~ 2 meV), from the FWHM of the Raman peak (indicated by the green double arrows).

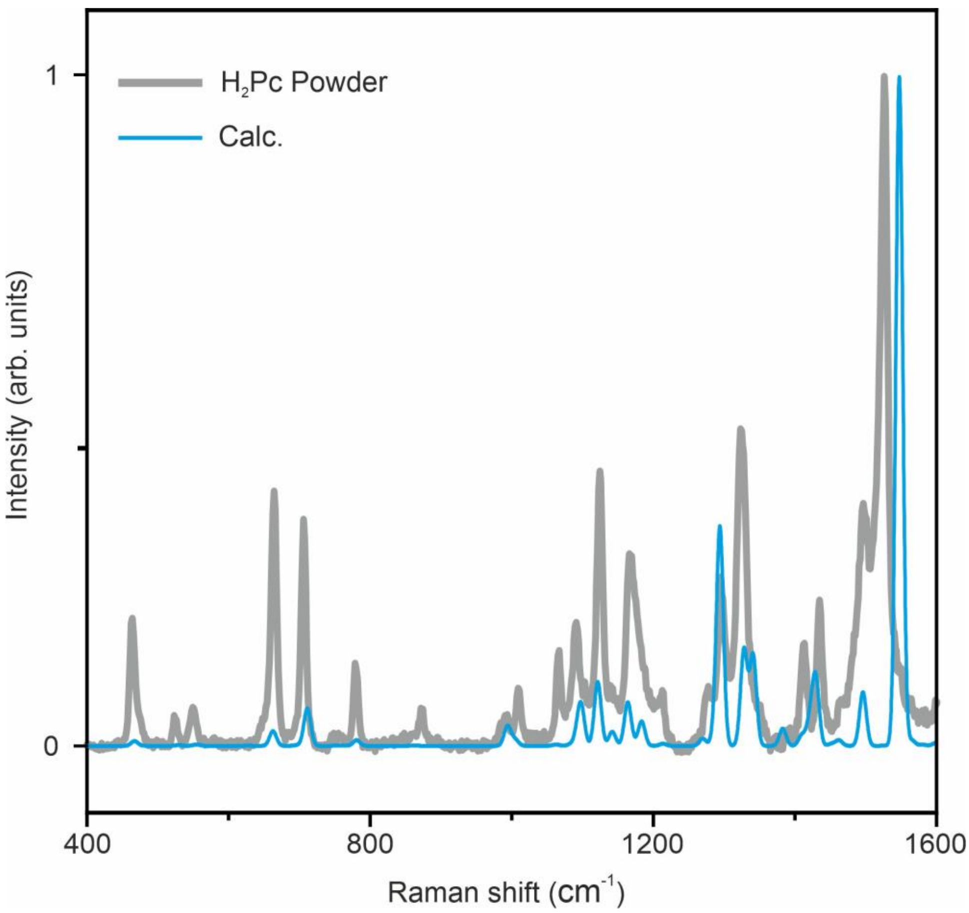

Supplementary Fig. 6: Comparison between the measured Raman spectrum from the powder of H_2Pc molecules (gray curve) and the calculated Raman spectrum for the ground electronic state (red curve). The Raman spectra were recorded with CW laser excitation (λ ~ 633 nm and laser power of 0.2 mW).

Section IV. UV-Vis absorption spectrum of H_2Pc molecules

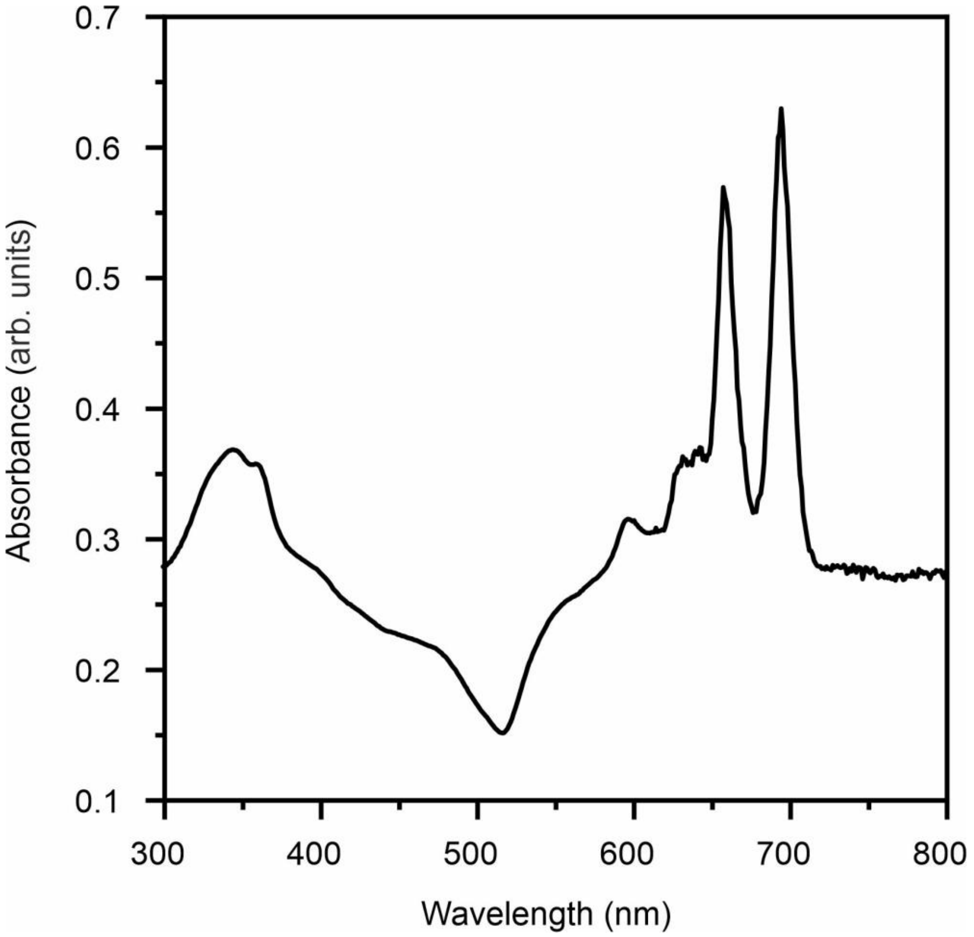

Supplementary Fig. 7: UV-Vis absorption spectrum of H_2Pc molecules dissolved in Chlorobenzene solvent. The concentration of the molecules in the solvent was ~ 0.1 mg/ml. The UV-Vis spectrum of H_2Pc molecules show two strong absorption regions, one in the UV region at about 300–400 nm (B band) and the other in the visible region at about 600–700 nm (Q band), including the characteristic splitting of the Q band into Q_x and Q_y transitions. The peak spectral positions of the Q_x and Q_y bands are red-shifted in the UV-Vis spectrum with respect to the single molecule TEPL spectrum due to the effect of the solvent.

References

1 Frisch, M. e. *et al. Gaussian 16, revision C. 01.* (Gaussian, Inc., Wallingford CT, 2016).

2 Becke, A. D. Density-Functional Thermochemistry .3. The Role of Exact Exchange. *J. Chem. Phys.* **98**, 5648-5652 (1993).

3 Baiardi, A., Bloino, J. & Barone, V. A general time-dependent route to Resonance-Raman spectroscopy including Franck-Condon, Herzberg-Teller and Duschinsky effects. *J. Chem. Phys.* **141**, 11 (2014).

4 Egidi, F., Boino, J., Cappell, C. & Barone, V. A Robust and Effective Time-Independent Route to the Calculation of Resonance Raman Spectra of Large Molecules in Condensed Phases with the Inclusion of Duschinsky, Herzberg-Teller, Anharmonic, and Environmental Effects. *J. Chem. Theory Comput.* **10**, 346-363 (2014).

5 Luo, Y. *et al.* What can single-molecule Fano resonance tell? *J. Chem. Phys.* **154**, 044309 (2021).

6 Imada, H. *et al.* Single-Molecule Investigation of Energy Dynamics in a Coupled Plasmon-Exciton System. *Phys. Rev. Lett.* **119**, 013901 (2017).
